# Effectiveness of balneotherapy in reducing pain, disability, and depression in patients with Fibromyalgia syndrome: a systematic review with meta-analysis

**DOI:** 10.1007/s00484-024-02732-3

**Published:** 2024-07-15

**Authors:** Héctor García-López, María Teresa García-Giménez, Esteban Obrero-Gaitán, Inmaculada Carmen Lara-Palomo, Adelaida María Castro-Sánchez, Raúl Romero-del Rey, Irene Cortés-Pérez

**Affiliations:** 1 Department of Nursing, Physical Therapy and Medicine, University of Almeria, Carr. Sacramento, s/n, La Cañada, Almería, 04120 Spain; 2 Department of Health Sciences, University of Jaén, Campus Las Lagunillas s/n, Jaén, 23071 Spain

**Keywords:** Balneotherapy, Depression, Disability, Fibromyalgia syndrome, Pain, Spa therapy

## Abstract

Balneotherapy, using heated natural mineral waters at 36–38 °C, presents a comprehensive treatment approach for Fibromyalgia Syndrome (FMS). This study aims to assess the effect of balneotherapy in reducing pain intensity, disability, and depression in patients with FMS. We want to assess this effect at just four time-points: immediately at the end of the therapy, and at 1, 3, and 6 months of follow-up. Following PRISMA guidelines, we conducted an aggregate data meta-analysis, registered in PROSPERO CRD42023478206, searching PubMed Medline, Science Direct, CINAHL Complete, Scopus, and Web of Science until August 2023 for relevant randomized controlled trials (RCTs) that assess the effect of balneotherapy on pain intensity, disability, and depression in FMS patients. Methodological quality was assessed using the Cochrane methodology, and the pooled effect was calculated using Cohen’s standardized mean difference (SMD) and its 95% confidence interval (95% CI) in a random-effects model. Sixteen RCTs were included in the meta-analysis. Balneotherapy is effective in reducing pain intensity (SMD − 1.67; 95% CI -2.18 to -1.16), disability (SMD − 1.1; 95% CI -1.46 to -0.7), and depression (SMD − 0.51; 95% CI -0.93 to -0.9) at the end of the intervention. This effect was maintained at 1, 3, and 6 months for pain intensity and disability. Balneotherapy improves both pain intensity and disability in patients with FMS, providing evidence that its positive effects are sustained for up to 6 months of follow-up. Nevertheless, it is important to note that the improvement in depression varies across different temporal phases.

**Supplementary Information:**

The online version contains supplementary material available at 10.1007/s00484-024-02732-3.

## Introduction

Fibromyalgia syndrome (FMS), historically viewed as an enigmatic clinical construct, has evolved toward a more defined recognition as a syndrome with internationally validated diagnostic criteria (Inanici and Yunus 2004). The World Health Organization (WHO) classifies FMS as a rheumatic disorder characterized by widespread musculoskeletal pain, fatigue, and sleep and mood disturbances (Wolfe et al. 2011). Although its etiology remains at the center of intense scientific debates, it is suggested that it lies in central sensitization, indicating an anomaly in pain processing at the central level, which manifests in a variety of comorbidities and complex pathophysiology (Sluka and Clauw 2016; Arnold et al. 2019). Furthermore, the interplay of genetic, biological, psychological, and environmental factors offers a vast field of study in both clinical and basic research (Buskila and Sarzi-Puttini 2006).

Globally, the prevalence of FMS is estimated to be between 2% and 4%, predominantly affecting women at a 7:1 ratio (Clauw 2014). In Europe, prevalence varies, reported between 2% and 6%, with a specific prevalence in countries such as France, Germany, and Spain ranging from 1.4 to 5.6% (Branco et al. 2010; Kocyigit and Akyol 2022). The economic burden associated with FMS is substantial, reflecting healthcare costs that vary widely due to the heterogeneity of symptoms and treatments (Berger et al. 2007). According to a 2022 study, annual direct healthcare costs for patients with FMS in the United States ranged from $1,750 to $35,920 per patient, and in Europe between $1,250 and $8,504, with medications being one of the largest contributors to total direct expenses (D’Onghia et al. 2022).

The diagnosis of FMS represents a clinical challenge due to the variability and overlap of its symptoms, including persistent pain, stiffness, fatigue, sleep disorders, cognitive impairment, and psychiatric manifestations (Wolfe et al. 1990; Häuser et al. 2015). The criteria of the American College of Rheumatology of 1990, focusing on widespread pain and tender points (Wolfe et al. 1990), have been revised to improve their applicability and accuracy, introducing the Widespread Pain Index and the Symptom Severity Scale in the 2010 and 2016 revisions (Wolfe et al. 2010, 2016).

Treatment for FMS aims to alleviate pain and improve patient functionality and quality of life (Sarzi-Puttini et al. 2011). Treatment regimens include drugs such as anticonvulsants, serotonin and norepinephrine reuptake inhibitors, and tricyclic antidepressants (Häuser et al. 2014; Rico-Villademoros et al. 2020), complemented by nonpharmacological therapies such as aerobic exercises (Newcomb et al. 2011), cognitive behavior therapy (Bernardy et al. 2010), and nutritional therapy (Silva et al. 2019). Balneotherapy, using heated natural mineral waters at 36–38 °C, offers a multifaceted treatment approach for FMS (Guidelli et al. 2012). This therapy goes beyond simple immersion in thermal waters, integrating gas treatments (like CO2 or sulfur), mud applications, physical exercises, and massages, all aimed at improving well-being and diminishing musculoskeletal pain (Verhagen et al. 2008; de Oliveira et al. 2023). The effectiveness of balneotherapy is due to the synergistic effect of water temperature and hydrostatic pressure, which improve muscle tone and reduce pain and stiffness (Silva et al. 2023). Additionally, the heat of water stimulates the production of hormones such as adrenocorticotropic hormone, cortisol, prolactin, and growth hormone, thus expanding its therapeutic scope (Mooventhan and Nivethitha 2014). Furthermore, balneotherapy has shown effectiveness in countering oxidative stress, a key factor in the pathogenesis of FMS, by balancing free radicals and antioxidants, thus reducing cellular damage, as supported by various in vitro and in vivo studies (Burguera et al. 2014; Fioravanti et al. 2015; Çetinkaya et al. 2020).

The existing literature on balneotherapy in the treatment of FMS presents certain limitations that warrant attention. Potential bias due to the limited number of randomized controlled trials (RCTs) and patients involved has been highlighted in the research by Naumann and Sadaghiani (2014). Other relevant reviews in this field have examined the effects of balneotherapy field (McVeigh et al. 2008; Fraioli et al. 2013). However, these reviews were limited by a restricted search period and language restrictions, excluding more recent and potentially relevant studies. Additionally, the most recent systematic review with meta-analysis (Cao et al. 2021) did not incorporate all the studies that met the inclusion criteria as reported by the authors, which could limit the breadth and applicability of its conclusions. Updated and expanded research, including all relevant studies, is essential for a more comprehensive understanding of the effects of balneotherapy in the treatment of FMS. The aim of this systematic review with meta-analysis is to assess the effect of balneotherapy in reducing pain intensity, disability, and depression in patients with FMS at the end of the intervention, and complementarily at 1, 3, and 6 months post-therapy.

## Materials and methods

### Protocol registration and study design

This systematic review with meta-analysis was carried out following the PRISMA 2020 Statement (Page et al. 2021) and the Cochrane Handbook for Systematic Reviews of Interventions (Higgins et al. 2019). This review protocol was registered in the PROSPERO database (CRD42023478206).

### Literature search

Two authors, independently, carried out a literature search in PubMed Medline, Science Direct, CINAHL Complete, Scopus, and Web of Science. Additionally, the authors searched the reference lists of previous reviews and original studies, and in complementary sources such as gray literature, document experts, congress abstracts, and proceedings. Duplicates found in the different databases were meticulously removed using the Rayyan QCRI software to maintain a concise and relevant selection of studies. The Boolean operators ‘AND’ and ‘OR’ were used to refine the search, combining the following MeSH terms and keywords: “balneology”, “hydrotherapy”, “spa therapy”, “water therapy”, “aquatic therapy”, “thermal therapy”, “mineral baths”, “balneotherapy”, “medicinal water” for therapeutic interventions and “Fibromyalgia”, “fibrositis”, “myofascial pain syndromes”, “chronic widespread pain” for the condition of interest. No restrictions related to Language and publication date filters were applied. The search strategies used in each database are displayed in Online Resource 1.

### Study selection criteria

Two authors, independently, screened the retrieved studies of each database. A first screening was made by title/abstract by each author. Each author reviewed in detail all potential inclusion records screened by the other author, with the aim of ensuring that all ineligible studies were correctly excluded. Any doubts were arbitrated by a third author.

To include a study in this review, it should have met each of the following inclusion criteria: (1) RCT or RCT pilot study; (2) that assesses the effectiveness of balneotherapy; (3) in comparison with controls (usual care or other physical therapy approaches); (4) on pain intensity, disability, and depression; (5) in patients with FMS. Another inclusion criteria is that the included studies provided the necessary data to perform the meta-analysis (sample size, mean, and standard deviation post-treatment of the variable of interest). As exclusion criteria, we propose removing studies with a sample comprised not exclusively of patients with FMS.

### Data extraction

Two authors, independently, extracted the data of interest from the included studies using a standardized data collection form. In accordance with the Cochrane recommendations (Higgins et al. 2019), data management was organized in the summary Table 1. We collected general data of the study, such as the publication date, authorship, funding source, country, demographic data of the participants (number, sex, age, and groups). Additionally, we collected data related to the balneotherapy and control interventions (type, duration, and frequency). Finally, we retrieved quantitative data from variables (mean and standard deviation), tests administered, and follow-up periods. In some studies, which reported a range instead of standard deviation, we estimated the standard deviation using the transformations contained in the Cochrane Handbook for Systematic Reviews of Interventions.

**Table 1:** Characteristics of the studies included in the review and interventions

	EG intervention		CG intervention			
Study	EG	EG protocol and frequency	CG	CG protocol and frequency	**Outcomes**	Qualitative findings and clinical relevance
Ardiç et al., 2007 (Turkey) *Fund: Yes* *N* = 21 F/M: 21/0	*N* = 12 Age (years): 43.5 ± 10.2	Intervention: BT *(36ºC).* Water composition: *Bicarbonate, chloride, sulfate, fluoride, calcium, magnesium, sodium, potassium.* Frequency: 3 weeks, 5 sessions per week, 20 min per session. Adverse effects: *NA.*	*N* = 9 Age (years): 48.8 ± 8.9	Intervention: UPT. Frequency: 3 weeks.	Pain (VAS) Disability (FIQ) Depression (BDI)	In post-immediate assessment, statistically significant differences favor BT in pain (*p* < 0.001), disability (*p* < 0.05), and depression (*p* < 0.001). BT effect was clinically relevant for pain, disability and depression
Bağdatlı et al., 2015 (Turkey) *Fund: NA* *N* = 70 F/M: 70/0	*N* = 35 Age (years): 45.2 ± 9.1	Intervention: BT *(20 min; 38ºC)* and mud-pack therapy *(20 min; 45ºC*). Water composition: *NA*. Frequency: 2 weeks, 5 sessions per week, 40 min per session. Adverse effects: *No.*	*N* = 35 Age (years): 42.8 ± 9.6	Intervention: UPT. Frequency: 2 weeks.	Pain (VAS) Disability (FIQ) Depression (BDI)	In post-immediate assessment and 1 month, statistically significant differences favors BT in pain (*p* = 0.002, *p* = 0.002), FIQ (*p* < 0.001, *p* = 0.006). No differences were found between therapies at 3 months for pain, disability, and depression. BT effect was clinically relevant for pain and disability in all time-point assessments
Buskila et al. 2001 (Israel) *Fund: NA* *N* = 48 F/M: 48/0	*N* = 24 Age (years): 54.6 ± 8.4	Intervention: BT *(37ºC).* Water composition: *Sulfu*r. Frequency: 10 days, 20 min per session. Adverse effects: *NA.*	*N* = 24 Age (years): 54.3 ± 8.0	Intervention: UPT. Frequency: 10 days.	Pain (VAS) Disability (FIQ) Depression (VAS)	No statistically significant differences were found between groups in post-immediate, and 1 and 3 months on pain, disability, and depression. BT effect was clinically relevant for pain just to the end of the intervention.
Dönmez et al. 2005 (Turkey) *Fund: NA* *N* = 29 F/M: 29/0	*N* = 16 Age (years): 43.3 ± 7.5	Intervention: BT *(20* min; *36* ± 1*ºC)*, and pressured shower *(15* min; *37ºC)* or massage *(15* min) every day alternately. Water composition: *Sodium, chlorine, bicarbonate, and fluoride.* Frequency: 2 weeks, 6 sessions per week, 35 min per session. Adverse effects: *NA.*	*N* = 13 Age (years): 43.1 ± 6.9	Intervention: UPT. Frequency: 2 weeks.	Pain (VAS) Disability (FIQ) Depression (BDI)	BT effect was statistically significant and clinically relevant for pain (*p* < 0.001), disability (*p* = 0.003), and depression (*p* = 0.002) just to the end of the intervention, and at 1, 3, and 6 months. In control group, only statistical improvements were found on pain (*p* = 0.024)
Evcik et al., 2002 (Turkey) *Fund: NA* *N* = 42 F/M: 31/11	*N* = 22 Age (years): 42.0 ± 6.8	Intervention: BT *(36ºC).* Water composition: *Sodium, bicarbonate, and sulfate.* Frequency: 3 weeks, 5 sessions per week, 20 min per session. Adverse effects: *No.*	*N* = 20 Age (years): 41.5 ± 7.1	Intervention: UPT. Frequency: 3 weeks.	Pain (VAS) Disability (FIQ) Depression (BDI)	BT was immediately statistically significant in reducing pain (*p* < 0.001), disability (< 0.001) and depression (< 0.01) and at 6 months in pain (*p* < 0.05) and disability (*p* < 0.05). No differences were found in control group for any variable. BT effect was clinically relevant for pain just to the end of the intervention and at 6 months for pain and disability
Fioravanti et al. 2007 (Italy) *Fund: NA* N *=* 80 F/M: 78/2	*N* = 40 Age (years): 46.2 ± 10.5	Intervention: Mud packs (15 min; 40–45ºC), and BT (10 min; 37–38ºC). Water composition: *Sulfate.* Frequency: 2 weeks, 6 sessions per week, 25 min per session. Adverse effects: *No.*	*N* = 40 Age (years): 48.6 ± 9.4	Intervention: UPT. Frequency: 2 weeks.	Pain (VAS) Disability (FIQ)	BT was immediately statistically significant in reducing pain (*p* < 0.0001), disability (< 0.001) and depression (< 0.01) and at 6 months in pain (*p* < 0.0001) and disability (*p* < 0.001). BT effect was clinically relevant for pain just to the end of the intervention and at 3 months for pain and disability
Fioravanti et al. 2018 (Italy) *Fund: Yes* N *=* 100 F/M: 95/5	*N* = 50 Age (years): 56.2 ± 8.7	Intervention: BT with highly mineralized sulfate water *(15 min; 36ºC)*, and bed rest *(15 min).* Water composition: *Sulfate.* Frequency: 2 weeks, 6 sessions per week, 30 min per session. Adverse effects: *Pain/stiffness, asthenia, headache, and insomnia.*	*N* = 50 Age (years): 55.9 ± 6.6	Intervention: Placebo BT with tap water *(15 min; 36ºC)*, and bed rest *(15 min).* Frequency: 2 weeks, 6 sessions per week, 30 min per session.	Pain (VAS) Disability (FIQ)	Statistically significant differences favor BT in post-immediate and at 3 and 6 months in reducing pain (*p* = 0.01, *p* = 0.0001 and *p* = 0.001) and disability (*p* = 0.05. *p* = 0.001 and *p* = 0.001). BT effect was clinically relevant for pain just to the end of the intervention and at 3 and 6 months for pain and disability
Kesiktas et al. 2011 (Turkey) *Fund: NA* N *=* 56 F/M: 56/0	EG_1_: *N* = 16 Age (years): 46.9 ± 9.2 EG_2_: *N* = 20 Age (years): 42.9 ± 8.3	EG_1_ intervention: PT modalities and BT *(20 min; 37–38ºC).* Water composition: *Carbon dioxide, bicarbonate, sulfate, fluoride, calcium, magnesium, sodium, and potassium.* Frequency: 2 weeks, 6 sessions per week, 30 min per session. Adverse effects: *NA.* EG_2_ intervention: PT modalities and hydrotherapy *(20 min; 37ºC).* Water composition: *Idem*. Frequency: 2 weeks, 6 sessions per week, 30 min per session.	*N* = 20 Age (years): 44.7 ± 8.8	Intervention: PT modalities: TENS *(15 min)*, ultrasound *(6 min; 1.5 W/cm*^*2*^*)*, and infrared heat lamp *(15 min; 50 cm, 250 W).* Frequency: 3 weeks, 5 sessions per week, 36 min per session.	Pain (VAS) Depression (BDI)	Statistically significant differences favor BT in post-immediate and at 6 months in reducing pain (*p* < 0.05) and depression (*p* < 0.05). BT effect was clinically relevant for pain and depression
Koçyiǧit et al., 2016 (Turkey) *Fund: NA* N *=* 56 F/M: 56/0	*N* = 31 Age (years): 42.5 ± 9.9	Intervention: Education *(20 min)* and BT *(20 min; 34,8ºC).* Water composition: *Sodium, potassium, ammonium, magnesium, calcium, manganese, iron, fluoride, chloride, bromur, iodide, nitride, nitrate, sulfate, bicarbonate, sulfur, and phosphate.* Frequency: 4 weeks, 5 sessions per week, 40 min per session. Adverse effects: *NA.*	*N* = 30 Age (years): 41.8 ± 10.5	Intervention: Education. Frequency: at the beginning of the treatment, 15th day, 1st month, 3rd month, and 6th month *(20 min)*.	Pain (VAS) Disability (FIQ)	BT and control interventions were immediately and at 1, 3, and 6 months statistically significant in reducing pain (*p* < 0.001) and disability (< 0.001). BT and control were clinically relevant for pain and depression reduction
Kurt et al. 2016 (Turkey) *Fund: No* N *=* 120 F/M: 120/0	EG_1_: *N* = 40 Age (years): 38.1 ± 10.9 EG_2_: *N* = 40 Age (years): 35.1 ± 11.6	EG_1_ intervention: *BT (20 min;* 42 ± 1*ºC).* Water composition: *Bicarbonate, sulfur, magnesium, calcium, chlorine, and fluorine.* Frequency: 3 weeks, 5 sessions per week, 20 min per session. EG_2_ intervention: *BT (20 min;* 42 ± 1*ºC)*, and exercise *(20–35 min).* Water composition: *Idem*. Frequency: 3 weeks, 5 sessions per week, 45–55 min per session. Adverse effects: *NA.*	*N* = 40 Age (years): 41.9 ± 12.8	Intervention: Exercise, which included stretching and strengthening exercises for the cervical, thoracic, and lumbar muscles. Frequency: 3 weeks, 5 sessions per week, 25–35 min per session.	Disability (FIQ) Depression (BDI)	Groups that received BT showed statistically significant reduction in post-immediate and 3 months in disability (*p* < 0.001 and *p* < 0.001) and depression (*p* < 0.001 and *p* = 0.002). BT was clinically relevant in reducing disability and depression
Maindet et al. 2021 (France) *Fund: Yes* N *=* 218 F/M: 198/20	*N* = 110 Age (years): 50.4 ± 8.9	Intervention: BT *(hydromassage baths, hydro-mineral mud applications, body jet showers, water affusion massages)* and collective exercise in a mineral water pool. Water composition: *NA.* Frequency: 3 weeks, 6 sessions per week, 120 min per session. Adverse effects: *No.*	*N* = 108 Age (years): 49.2 ± 8.8	Intervention: UPT. Frequency: 3 weeks.	Pain (VAS) Disability (FIQ) Depression (HADS)	Statistically significant differences favor BT at 3 and 6 months in reducing pain (*p* = 0.013) and depression (*p* = 0.05) were showed. BT effect was clinically relevant for pain reduction.
Neumann et al. 2001 (Israel) *Fund: NA* *N* = 48 F/M: 48/0	EG: *N* = 24 Age (years): 54.6 ± 8.4	Intervention: BT *(20 min; 37ºC).* Water composition: *Sulfur.* Frequency: 10 days, every day, 20 min per session. Adverse effects: *NA.*	*N* = 24 Age (years): 54.3 ± 8.0	Intervention: UPT. Frequency: 10 days.	Pain (VAS)	The group that received BT showed a statistically significant reduction in post-immediate and at 1 and 6 months in pain (*p* = 0.0002). BT effect was clinically relevant for pain reduction
Özkurt et al., 2012 (Turkey) *Fund: NA* N *=* 50 F/M: 50/0	*N* = 25 Age (years): 50.8 ± 6.0	Intervention: BT *(20 min; 36* ± 1*ºC ºC).* Water composition: *Sodium, chloride, and calcium.* Frequency: 2 weeks, 12 sessions per week *(2 times/day for 6 days)*, 20 min per session. Adverse effects: *NA.*	*N* = 25 Age (years): 46.9 ± 8.8	Intervention: UPT. Frequency: 2 weeks.	Pain (VAS) Disability (FIQ) Depression (BDI)	The group that received BT showed a statistically significant reduction in post-immediate and at 1 and 3 months in pain (*p* < 0.001), disability (*p* < 0.001), and depression (*p* < 0.001). No statistically significant improvements were found in the control group. BT effect was clinically relevant for pain, disability, and depression reduction
Pérez-Fernández et al. 2019 (Spain) *Fund: NA* N *=* 50 F/M: 48/2	EG: *N* = 25 Age (years): 52.4 ± 8.6	Intervention: BT *(30 min; 38ºC).* Water composition: *Sodium bicarbonate, lithium, fluoride and silicates.* Frequency: 4 weeks, 3 sessions per week, 30 min per session. Adverse effects: *NA.*	*N* = 25 Age (years): 53.4 ± 11.3	Intervention: UPT. Frequency: 4 weeks.	Disability (FIQ)	BT was immediately and at 1 and 3 months statistically significant in reducing disability (*p* < 0.001). Statistically significant differences favoring balneotherapy (*p* < 0.05) were shown. BT effect was clinically relevant for disability reduction
Yurtkuran et al., 1996 (Turkey) *Fund: NA* N *=* 40 F/M: 37/3	*N* = 20 Age (years): 37.5	Intervention: BT *(20 min; 37ºC)*, and relaxation exercises. Water composition: *Bicarbonate, chloride, fluoride, sulfate, calcium, magnesium, sodium, potassium, and lithium.* Frequency: 2 weeks, 5 sessions per week, 20 min per session. Adverse effects: *NA.*	*N* = 20 Age (years): 33.4	Intervention: Relaxation exercises. Frequency: 2 weeks, 5 sessions per week.	Pain (VAS)	The group that received BT showed statistically significant reduction in post-immediate and at 1 month in pain (*p* = 0.001, *p* = 0.001). BT effect was clinically relevant for pain reduction
Zijlstra et al. 2005 (Tunisia) *Fund: Yes* N *=* 134 F/M: 128/6	EG: *N* = 58 Age (years): 48.0	Intervention: BT, exercise, patient education, and recreational activities. Water composition: *NA.* Frequency: 15 days, 4 sessions per week, 180 min per session. Adverse effects: *No.*	*N* = 76 Age (years): 47.0	Intervention: UPT. Frequency: 15 days.	Pain (VAS) Disability (FIQ) Depression (FIQ)	Statistically significant differences favor BT were found at 3reducing disability (p *≤* 0.01), but not at 6 months. No statistically significant reduction was found in pain and depression. BT effect was not clinically relevant for disability reduction.

*Abbreviations* BDI, Beck Depression Inventory; BT, Balneotherapy; CG, Control Group; EG, Experimental Group; F, Female; Fund, Funding; FIQ, Fibromyalgia Impact Questionnaire; HADS, Hospital Anxiety and Depression Scale; M, Males; Min, minutes; N, number of participants; NA, not available; T, Time; UPT, Usual pharmacological treatment; VAS, Visual Analogue Scale

### Variables and tests

The variables of interest in this systematic review with meta-analysis were pain intensity, disability, and depression. Pain intensity, defined as the subjective unpleasant perception by the patients, was assessed with data from Visual Analogue Scale (VAS), a straight horizontal line orientated from the left (no pain) to the right (worst pain) (Price et al. 1983). The Fibromyalgia Impact Questionnaire (FIQ) was used to determine the disability or the alteration in functional status of the patients. FIQ is a 20-item, self-administered instrument that measures physical functioning, work status, depression, anxiety, sleep, pain, stiffness, fatigue, and wellbeing (Burckhardt et al. 1991). For depression, we used data from the Beck Depression Inventory (BDI) and the FIQ-depression dimension. BDI is a test comprised of 21 questions that quantify the level of depression in the last week. Lower scores indicate no or low depression (Beck et al. 1996).

### Risk of bias assessment

The risk of bias assessment for all RCTs was performed using the Cochrane Collaboration’s risk of bias tool (CRoB) (Higgins et al. 2019), independently applied by two reviewers. Agreeing with Armijo-Olivo et al., CrOB is the most appropriate measurement to assess the risk of bias in physiotherapy studies (Armijo-Olivo et al. 2015a). Studies were categorized as having low, high, or unclear risk of bias based on the following domains: random sequence generation, allocation concealment, blinding of participants and personnel, blinding of outcome assessment, completeness of outcome data, selective reporting, and other biases. When discrepancies arose, a third evaluator was consulted to reach consensus, ensuring a systematic and objective assessment of the risk of bias in the review.

Additionally, the quality of evidence was assessed using the Grading of Recommendations Assessment, Development, and Evaluation (GRADE) tool (Atkins et al. 2004) and the GRADE checklist of Meader (Meader et al. 2014). The quality of evidence was determined according to five items: risk of bias in each study, imprecision, inconsistency, indirect evidence, and publication bias. Inconsistency was analyzed by estimating the heterogeneity of the findings. Imprecision was assessed by the number of studies included, the number of participants in each meta-analysis, and the number of participants per study (large imprecision < 100 participants or < 5 studies; moderate imprecision = 100–300 participants and 5–10 studies; and low imprecision = > 300 participants or > 10 studies). Indirect evidence appears in studies in which the results were measured indirectly. Combining these items, quality of evidence can be high (if findings are robust), moderate (when a future study can change the current findings), low (poor level of confidence), and very low (if four or more items are not met). The quality of evidence was downgraded one level for each item that did not meet the specified criteria. The risk of bias and quality of evidence assessment was carried out by two authors, and disagreements were resolved by a third author.

### Statistical analysis

The meta-analysis was carried out using the Comprehensive Meta-Analysis version 4.0 (Biostat, Inc.) (Borenstein et al. 2009) by two expert authors in meta-analyses. We carried out a meta-analysis of aggregate data (a statistical method that combines information from published, full-text studies to estimate the outcome of interest) (Blettner et al. 1999; McGrath et al. 2019). To generalize the findings, we used a random-effects model by Dersimonian and Laird (DerSimonian and Laird 1986; Cooper et al. 2009). Pooled effect was calculate using the Cohen standardized mean difference (SMD) and its 95% confidence interval (95% CI). Effect sizes could be null (SMD 0), low (SMD 0.1–0.3), medium (SMD 0.4–0.7) and large (SMD *≥* 0.8) (Cohen 1977; Faraone 2008). Meta-analyses were graphically displayed in the forest plots (Rücker and Schwarzer 2021). Publication bias was estimated using the visualization of the funnel plot, the Egger p-value, and the trim-and-fill estimation. Asymmetric funnel plot, Egger *p* < 0.1 and > 10% of variation in effect after trim-and-fill estimation indicates the presence of publication bias. Variations > 10% between the original and the adjusted effect size suggest a risk of publication bias and downgrade the quality of evidence one level, even if the funnel plot is symmetric (Rothman et al. 2015). Statistical heterogeneity was calculated through the degree of inconsistency of Higgins (I^2^), the Q-test, the degree of freedom, and its p-value (*P* < 0.05 indicates possible heterogeneity). According to I^2^, heterogeneity can be null (I^2^ 0%), low (I^2^ 5–25%), moderate (I^2^ 25–50%), and large (I^2^ > 50%) (Higgins et al. 2002; Higgins and Thompson 2002).

Meta-analyses were performed according to 4 subgroups, each one for a different time-point: just at the end of the intervention, and at 1, 3, and 6 months of follow-up since the end of the intervention. A sensitivity analysis (leave-one-out method) was carried out to assess the contribution of each study to the global effect size.

## Results

### Study selection

**Fig. 1:**
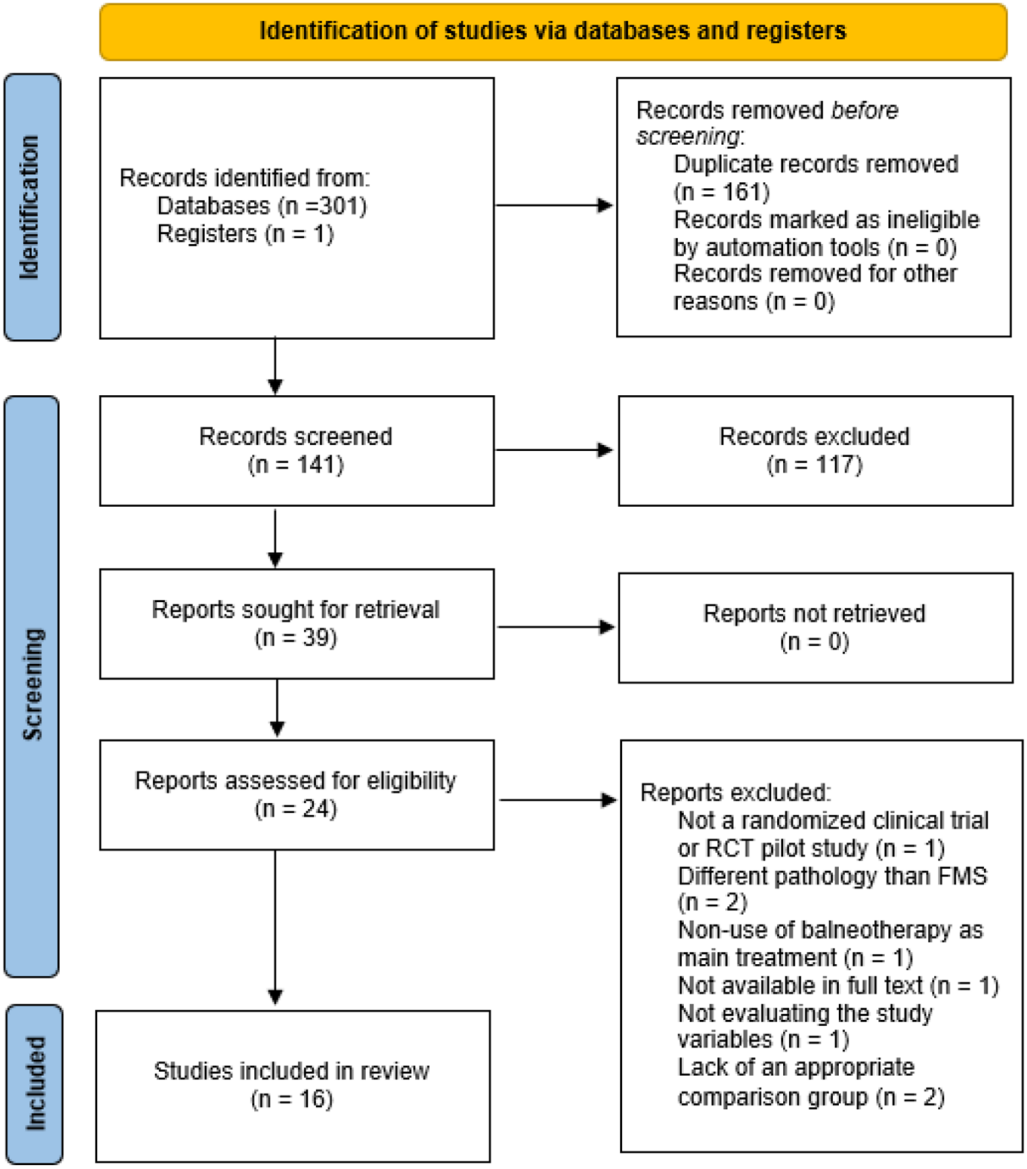
PRISMA flow diagram

Three hundred and one references were recovered from the databases (PubMed Medline *n* = 36, WOS *n* = 45, Elsevier *n* = 61, SCOPUS *n* = 113, and CINAHL Complete *n* = 46). After eliminating duplicates, 141 references were screened by title/abstract, and 117 studies were excluded for not being relevant. Following this process, 24 studies underwent a full-text analysis, excluding 8 studies for not meeting inclusion criteria: different study design from RCT or RCT pilot study (*n* = 1), sample with a different pathology from FMS (*n* = 2), non-use of balneotherapy as the main treatment (*n* = 1), not available in full text (*n* = 1), not evaluating the study variables (*n* = 1), and lack of an appropriate comparison group (*n* = 2). Finally, 16 studies were included in the present systematic review (Yurtkuran and Celiktas 1996; Buskila et al. 2001; Neumann et al. 2001; Evcik et al. 2002; Zijlstra et al. 2005; Dönmez et al. 2005; Ardıç et al. 2007; Fioravanti et al. 2007, 2018; Kesiktas et al. 2011; Özkurt et al. 2012; Bağdatlı et al. 2015; Kurt et al. 2016; Koçyiǧit et al. 2016; Pérez-Fernández et al. 2019; Maindet et al. 2021). Figure 1 (the PRISMA flow diagram) illustrates the study selection process.

### Characteristics of the studies included in the systematic review

The studies included in the review were carried out in countries such as France, Israel, Italy, Spain, Tunisia, and Turkey between 1996 and 2021, providing data from 1167 patients with FMS (96% women) with a mean age of 46.6 ± 8.8 years old. The intervention group (balneotherapy group) consisted of 608 patients, while the comparison group consisted of 559 patients. Regarding the characteristics of the balneotherapy intervention, thermal water was used at temperatures ranging from 34.8 to 42 ± 1ºC and applied for durations of 10 to 30 min. Regarding the mineral composition of the water, two studies used sulfur water(Buskila et al. 2001; Neumann et al. 2001) and two studies used sulfate water (Fioravanti et al. 2007, 2018). Most studies utilized water with various mineral components (Yurtkuran and Celiktas 1996; Evcik et al. 2002; Dönmez et al. 2005; Ardıç et al. 2007; Kesiktas et al. 2011; Özkurt et al. 2012; Kurt et al. 2016; Koçyiǧit et al. 2016; Pérez-Fernández et al. 2019). Three studies did not provide information on the mineral composition of the water used in their interventions (Zijlstra et al. 2005; Bağdatlı et al. 2015; Maindet et al. 2021). The number of sessions varied between studies, averaging 4.8 sessions (range of 3 to 12 sessions per week). Ten studies did not report data on the adverse effects of balneotherapy (Yurtkuran and Celiktas 1996; Buskila et al. 2001; Neumann et al. 2001; Dönmez et al. 2005; Ardıç et al. 2007; Kesiktas et al. 2011; Özkurt et al. 2012; Kurt et al. 2016; Koçyiǧit et al. 2016; Pérez-Fernández et al. 2019), while five studies reported no adverse effects associated with balneotherapy (Evcik et al. 2002; Zijlstra et al. 2005; Fioravanti et al. 2007; Bağdatlı et al. 2015; Maindet et al. 2021). One study reported the presence of pain/stiffness, asthenia, headache, and insomnia following the intervention (Fioravanti et al. 2018). Different interventions were carried out in the experimental group, ranging from isolated use of balneotherapy(Buskila et al. 2001; Neumann et al. 2001; Evcik et al. 2002; Ardıç et al. 2007; Özkurt et al. 2012; Kurt et al. 2016; Fioravanti et al. 2018; Pérez-Fernández et al. 2019) to its combination with patient education (Zijlstra et al. 2005; Koçyiǧit et al. 2016), physical exercise (Zijlstra et al. 2005; Kurt et al. 2016; Maindet et al. 2021), relaxation exercises (Yurtkuran and Celiktas 1996), pressure shower (Dönmez et al. 2005; Maindet et al. 2021), mud-pack (Fioravanti et al. 2007; Bağdatlı et al. 2015; Maindet et al. 2021), or physical therapy modalities (Kesiktas et al. 2011). Eleven of the studies continued with the usual pharmacological treatment in the control group (Buskila et al. 2001; Neumann et al. 2001; Evcik et al. 2002; Zijlstra et al. 2005; Dönmez et al. 2005; Ardıç et al. 2007; Fioravanti et al. 2007; Özkurt et al. 2012; Bağdatlı et al. 2015; Pérez-Fernández et al. 2019; Maindet et al. 2021). However, in 5 of the studies, a different intervention was carried out in the control group, including physical therapy modalities (Kesiktas et al. 2011), patient education (Koçyiǧit et al. 2016), physical exercise (Kurt et al. 2016), relaxation exercises (Yurtkuran and Celiktas 1996), or placebo (Fioravanti et al. 2018). Table 1 summarizes the characteristics of the studies included in the review and interventions, including duration, the number of sessions conducted per week, and the total duration of each session.

### Risk of bias assessment

Due to the nature of the included studies (Buskila et al. 2001; Neumann et al. 2001; Evcik et al. 2002; Zijlstra et al. 2005; Dönmez et al. 2005; Ardıç et al. 2007; Fioravanti et al. 2007, 2018; Kesiktas et al. 2011; Özkurt et al. 2012; Bağdatlı et al. 2015; Kurt et al. 2016; Koçyiǧit et al. 2016; Pérez-Fernández et al. 2019; Maindet et al. 2021), participant and personnel blinding was not considered, resulting in their classification as having an “unclear risk of bias” in this aspect. Three studies were classified as having a “high risk of bias” due to the absence of blinding for both participants (Zijlstra et al. 2005; Pérez-Fernández et al. 2019; Maindet et al. 2021) and for evaluators (Pérez-Fernández et al. 2019). However, nine studies successfully implemented blinding of the assessors (Yurtkuran and Celiktas 1996; Buskila et al. 2001; Neumann et al. 2001; Dönmez et al. 2005; Fioravanti et al. 2007, 2018; Özkurt et al. 2012; Bağdatlı et al. 2015; Kurt et al. 2016). Random sequence generation was implemented in twelve of the included studies (Evcik et al. 2002; Zijlstra et al. 2005; Dönmez et al. 2005; Fioravanti et al. 2007, 2018; Kesiktas et al. 2011; Özkurt et al. 2012; Bağdatlı et al. 2015; Kurt et al. 2016; Koçyiǧit et al. 2016; Pérez-Fernández et al. 2019; Maindet et al. 2021), thus classifying them as a “low risk of bias.” However, several studies (Yurtkuran and Celiktas 1996; Buskila et al. 2001; Neumann et al. 2001; Ardıç et al. 2007) did not clearly detail the randomization process. Allocation concealment was classified as “low risk of bias” in all studies (Neumann et al. 2001; Zijlstra et al. 2005; Dönmez et al. 2005; Fioravanti et al. 2007, 2018; Kesiktas et al. 2011; Özkurt et al. 2012; Bağdatlı et al. 2015; Koçyiǧit et al. 2016; Pérez-Fernández et al. 2019; Maindet et al. 2021) except for five studies (Yurtkuran and Celiktas 1996; Buskila et al. 2001; Evcik et al. 2002; Ardıç et al. 2007; Kurt et al. 2016) that failed to provide information on this item, thereby being classified as “unclear risk of bias”. All studies (Buskila et al. 2001; Neumann et al. 2001; Evcik et al. 2002; Zijlstra et al. 2005; Dönmez et al. 2005; Ardıç et al. 2007; Fioravanti et al. 2007, 2018; Kesiktas et al. 2011; Özkurt et al. 2012; Bağdatlı et al. 2015; Kurt et al. 2016; Koçyiǧit et al. 2016; Pérez-Fernández et al. 2019; Maindet et al. 2021) were classified as “low risk of bias” for incomplete outcome data, except one study that did not report information on sample loss (Yurtkuran and Celiktas 1996). All studies were also classified as “low risk of bias” regarding selective reporting of results (Yurtkuran and Celiktas 1996; Buskila et al. 2001; Neumann et al. 2001; Evcik et al. 2002; Zijlstra et al. 2005; Dönmez et al. 2005; Ardıç et al. 2007; Fioravanti et al. 2007, 2018; Kesiktas et al. 2011; Özkurt et al. 2012; Bağdatlı et al. 2015; Kurt et al. 2016; Koçyiǧit et al. 2016; Pérez-Fernández et al. 2019; Maindet et al. 2021). Online Resources 2 and 3 summarize the risk of bias assessment according to Cochrane Risk of Bias Tool.

### Main findings in quantitative synthesis

#### Pain intensity

**Fig. 2:**
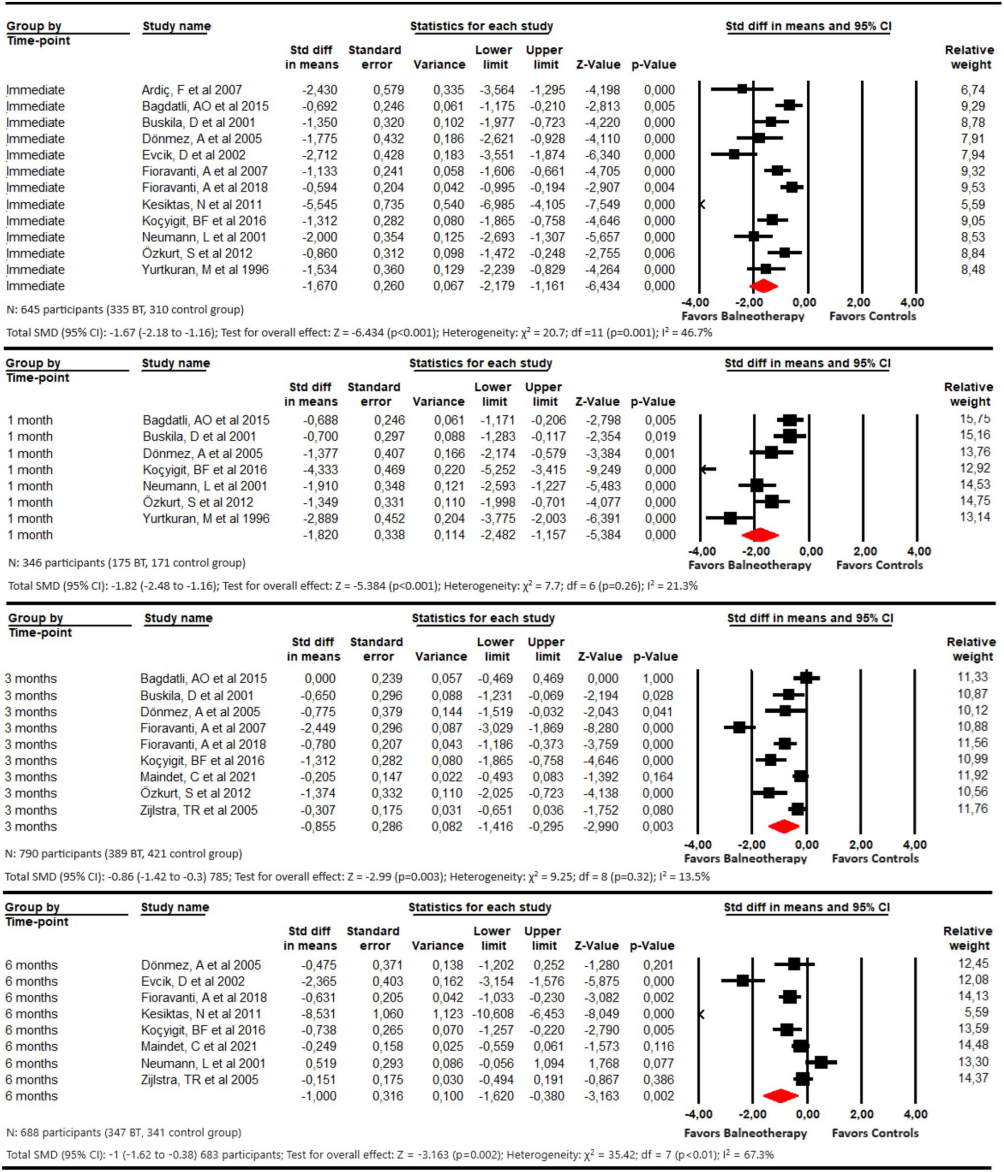
Forest plot for the comparison of pain immediately, at 1 month, at 3 months, and at 6 months, showing the effect favoring balneotherapy

For the assessment of pain intensity, we performed 4 meta-analyses. These meta-analyses (Fig. 2) showed that balneotherapy is effective (large effect) in reducing the pain intensity of patients with FMS with immediate effect (SMD − 1.67; 95% CI -2.18 to -1.16; p 0.04; I^2^ 46.7%; *p* < 0.001), and at 1 (SMD − 1.82; 95% CI − 2.48 to-1.16; *p* < 0.001; I^2^ 21.32%; p 0.26), 3 (SMD − 0.86; 95% CI -1.41 to -0.3; p 0.003; I^2^ 13.5%; p 0.32) and 6 months of follow-up (SMD − 1; 95% CI -1.62 to -0.38; p 0.002; I^2^ 67.3%; *p* < 0.01). Publication bias should be considered for the meta-analysis of at 1 and 6 months. In these meta-analyses, the trim-and-fill estimation suggests that the original effect could be underestimated due to publication bias (more details in Online Resource 4, 5, 6, 7, and 8).

#### Disability

**Fig. 3:**
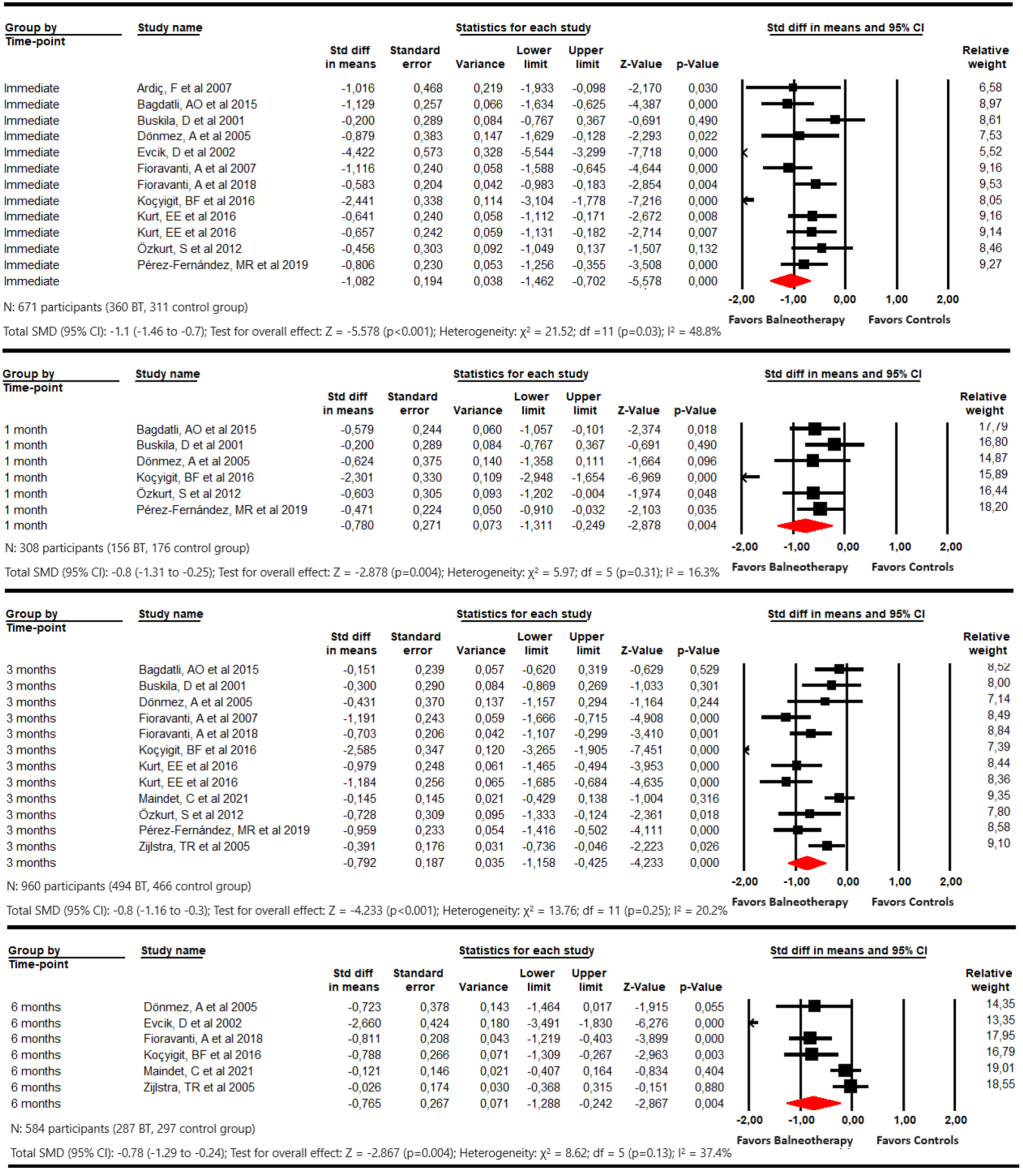
Forest plot for the comparison of disability immediately, at 1 month, at 3 months, and at 6 months, showing the effect favoring balneotherapy

Meta-analysis showed that balneotherapy is effective in reducing disability in these patients (Fig. 3) at the end of the intervention (SMD − 1.1; 95% CI -1.46 to -0.7; *p* < 0.001; I^2^ 48.8%; p 0.03). Additionally, the positive effect of balneotherapy was sustained at 1 (SMD − 0.78; 95% CI -1.31 to -0.25; p 0.004; I^2^ 16.3%; p 0.31), 3 (SMD − 0.8; 95% CI -1.16 to -0.43; *p* < 0.001; I^2^ 20.2%; p 0.25) and 6 months post intervention (SMD − 0.77; 95% CI -1.29 to -0.24; p 0.004; I^2^ 37.4%; p 0.13). Publication bias should be considered in all these findings, and trim-and-fill estimation indicates that the original pooled effect in each meta-analysis could be underestimated due to the presence of publication bias. Therefore, it is possible that the effect of balneotherapy was greater (more details in Online Resource 4, 9, 10, 11, and 12).

#### Depression

**Fig. 4:**
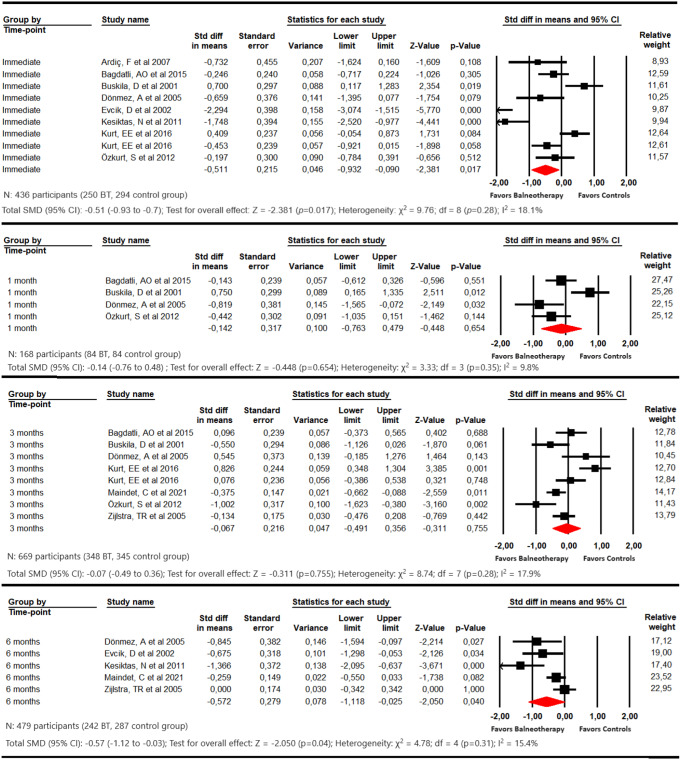
Forest plot for the comparison of depression immediately, at 1 month, at 3 months, and at 6 months, showing the effect favoring balneotherapy

Finally, for depression assessment, our meta-analysis reported statistically significant differences favoring balneotherapy at the end of the intervention (SMD − 0.51; 95% CI -0.93 to -0.9; p 0.017; I2 18.1%; p 0.28) (Fig. 4 and Online Resource 4). No differences were found at 1 SMD − 0.14; 95% CI -0.76 to 0.48; p 0.654; I^2^ 9.81%; p 0.35) and 3 months (SMD − 0.07; 95% CI -0.49 to 0.36; p 0.755; I^2^ 17.9%; p 0.28). However, at 6 months, statistically significant differences favoring balneotherapy were observed (SMD − 0.57; 95% CI -1.12 to -0.03; p 0.040; I^2^ 15.41%; p 0.31).

## Discussion

The purpose of this systematic review with meta-analysis was to assess the effect of balneotherapy in reducing pain intensity, disability, and depression in patients with FMS at the conclusion of the intervention, and additionally at 1, 3, and 6 months post-therapy. Our results revealed that balneotherapy is an effective therapy for reducing pain intensity, disability, and depression in patients with FMS. Furthermore, these effects on pain and disability may persist for up to 6 months. To date, this systematic review with meta-analysis is distinguished by incorporating the largest number of RCTs assessing the effectiveness of balneotherapy in patients with FMS. Two previous systematic reviews with meta-analysis were conducted (Naumann and Sadaghiani 2014; Cao et al. 2021) to address the efficacy of balneotherapy in FMS treatment. Specifically, Naumann and Sadaghiani (2014) included only 6 studies with data from 311 participants. The most recent systematic review with meta-analysis (Cao et al. 2021), similar to the present study, explored the effectiveness of balneotherapy in pain intensity, disability, and depression, encompassing a total of 11 studies and collecting data from 672 FMS patients. However, the current study augmented the meta-analysis by incorporating an additional 5 RCTs, yielding data from a total of 1167 participants with FMS, of whom 608 underwent balneotherapy just to the end of the therapy (post-immediate assessment). However, the total number of patients providing aggregated data for this meta-analysis must be classified according to all time points of assessment, namely at 3 months (9 RCTs, 685 enrolled, 389 receiving balneotherapy) and at 6 months (8 RCTs, 683 enrolled, 347 receiving balneotherapy). Additionally, this review updated the literature search up to August 2023, thereby including all relevant scientific literature for a broader generalization of the findings.

The first notable finding in our meta-analysis was the effectiveness of balneotherapy in reducing pain intensity in patients with FMS, yielding immediate effects (SMD = -1.67) (Yurtkuran and Celiktas 1996; Buskila et al. 2001; Neumann et al. 2001; Evcik et al. 2002; Dönmez et al. 2005; Ardıç et al. 2007; Fioravanti et al. 2007, 2018; Kesiktas et al. 2011; Özkurt et al. 2012; Bağdatlı et al. 2015; Koçyiǧit et al. 2016), as well as sustained at 1 month (SMD = -1.82) (Yurtkuran and Celiktas 1996; Buskila et al. 2001; Neumann et al. 2001; Dönmez et al. 2005; Özkurt et al. 2012; Bağdatlı et al. 2015; Koçyiǧit et al. 2016), 3 months (SMD = -0.86) (Buskila et al. 2001; Zijlstra et al. 2005; Dönmez et al. 2005; Fioravanti et al. 2007, 2018; Özkurt et al. 2012; Bağdatlı et al. 2015; Koçyiǧit et al. 2016; Maindet et al. 2021), and 6 months of follow-up (SMD = -1) (Neumann et al. 2001; Evcik et al. 2002; Zijlstra et al. 2005; Dönmez et al. 2005; Kesiktas et al. 2011; Koçyiǧit et al. 2016; Fioravanti et al. 2018; Maindet et al. 2021). Moreover, participants who received balneotherapy exhibited a decrease in pain intensity compared to the control group, comprising usual pharmacological treatment (Buskila et al. 2001; Neumann et al. 2001; Evcik et al. 2002; Dönmez et al. 2005; Ardıç et al. 2007; Özkurt et al. 2012; Maindet et al. 2021), patient education (Koçyiǧit et al. 2016), relaxation exercises (Yurtkuran and Celiktas 1996), or placebo balneotherapy (Fioravanti et al. 2018). These results align with previous meta-analyses (Naumann and Sadaghiani 2014; Cao et al. 2021). All therapies included in the control group have been recommended by various clinical practice guidelines for the treatment of this population (Rivera Redondo et al. 2021; Ariani et al. 2021; El Miedany et al. 2022). Consequently, balneotherapy emerges as a therapeutic option to consider for patients with FMS, considering its effects up to 6 months of follow-up. However, evidence suggesting pain relief beyond this period is lacking.

Regarding disability, one of the significant challenges faced by individuals with FMS is its substantial impact on various aspects of their lives, encompassing physical, psychological, and occupational factors, as evaluated through FIQ (Burckhardt et al. 1991). Our findings suggest that balneotherapy can significantly improve disability at the end of the intervention (SMD = -1.1) (Buskila et al. 2001; Evcik et al. 2002; Dönmez et al. 2005; Ardıç et al. 2007; Fioravanti et al. 2011, 2018; Özkurt et al. 2012; Bağdatlı et al. 2015; Kurt et al. 2016; Koçyiǧit et al. 2016; Pérez-Fernández et al. 2019). Additionally, the positive effect of balneotherapy was maintained at 1 (SMD = − 0.78) (Buskila et al. 2001; Dönmez et al. 2005; Özkurt et al. 2012; Bağdatlı et al. 2015; Koçyiǧit et al. 2016; Pérez-Fernández et al. 2019), 3 (SMD = -0.8) (Buskila et al. 2001; Zijlstra et al. 2005; Dönmez et al. 2005; Fioravanti et al. 2011, 2018; Özkurt et al. 2012; Bağdatlı et al. 2015; Kurt et al. 2016; Koçyiǧit et al. 2016; Pérez-Fernández et al. 2019; Maindet et al. 2021), and 6 months since the end of the therapy (SMD = -0.77) (Evcik et al. 2002; Zijlstra et al. 2005; Dönmez et al. 2005; Koçyiǧit et al. 2016; Fioravanti et al. 2018; Maindet et al. 2021). Moreover, participants underwent various interventions, including balneotherapy alone (Buskila et al. 2001; Evcik et al. 2002; Ardıç et al. 2007; Özkurt et al. 2012; Fioravanti et al. 2018; Pérez-Fernández et al. 2019) or in conjunction with other procedures such as pressure shower (Dönmez et al. 2005), mud packs (Fioravanti et al. 2007), education (Koçyiǧit et al. 2016), or other spa therapies (Maindet et al. 2021). These results, corroborated by the previous meta-analysis (Cao et al. 2021), are noteworthy given the complexity of this condition, which often necessitates a multimodal and personalized therapeutic approach (Kachaner et al. 2023). Additionally, a recent study reported a strong correlation between pain intensity and disability in patients with FMS (Fernandes et al. 2023).

In the assessment of depression, our meta-analysis revealed statistically significant differences favoring balneotherapy both at the end of the intervention (SMD = -0.51) and at 6 months (SMD = -0.57). These participants underwent balneotherapy either in isolation (Ardıç et al. 2007; Özkurt et al. 2012) or in combination with other techniques such as pressure shower or massage (Dönmez et al. 2005), physical therapy modalities (Kesiktas et al. 2011), and other spa therapies (Maindet et al. 2021). However, no differences were observed at 1 month (SMD = -0.14) and 3 months (SMD = -0.07). These findings are consistent with those reported by Cao et al. (2021), who also noted a statistically significant improvement in depression at 6 months after receiving balneotherapy. Interestingly, a strong positive correlation has recently been observed between pain intensity and depression, where a higher score on the VAS resulted in higher levels of depression (Fernandes et al. 2023). However, previous studies have suggested that this relationship is bidirectional (Zis et al. 2017; Viana et al. 2018; Bondesson et al. 2018), and pain is strongly associated with the onset and relapse of depression (Gerrits et al. 2014). Thus, it is plausible that the long-term pain relief observed in this meta-analysis in participants who received balneotherapy had a beneficial psychological effect on these patients, significantly reducing their depression levels. Despite this, in contrast to our findings, Cao et al. (2021) did not observe an immediate statistically significant effect. The primary distinction between the studies is that our research included six additional RCTs in the meta-analysis to analyze the immediate effect of balneotherapy on depression, which may have influenced the outcomes obtained in their meta-analysis.

The effects of balneotherapy are attributable to factors such as heat and mineral content (Fioravanti et al. 2011). Most of the included studies used water composed of various minerals (Yurtkuran and Celiktas 1996; Evcik et al. 2002; Dönmez et al. 2005; Ardıç et al. 2007; Kesiktas et al. 2011; Özkurt et al. 2012; Kurt et al. 2016; Koçyiǧit et al. 2016; Pérez-Fernández et al. 2019), while several studies used water containing only sulfur(Buskila et al. 2001; Neumann et al. 2001) or sulfate (Fioravanti et al. 2007, 2018). Given our results, there are no established criteria for these parameters, nor for the dosage and water temperature, which are critical in balneotherapy for patients with FMS. Overall, coupled with the absence of significant adverse effects, this establishes balneotherapy as a treatment option to consider for patients with FMS.

This systematic review boasts notable strengths, including incorporating a substantial number of RCTs, thus providing a comprehensive and diverse overview of the literature on balneotherapy in FMS. This approach results in an extensive sample of participants, enhancing the ability to generalize findings and draw robust conclusions. However, certain limitations of the study must be acknowledged. One important limitation to note is that this meta-analysis is based on aggregated data instead of individual data. Although it is relatively quick and low-cost to carry out, it has disadvantages, such as how it affects publication bias and the heterogeneity of study designs, protocols, and measurements, which can influence the pooled effect size. Additionally, the absence of sample size calculation to control for alpha risk in most RCTs and the low number of participants in some time-point assessments may limit the generalizability of our findings. The scarcity of long-term RCTs highlights the need to explore the behavior of the results during that period. The inability to blind participants and therapists, resulting in a high risk of performance bias, can affect the accuracy of the balneotherapy’s effect, potentially leading to an overestimation of the findings (Armijo-Olivo et al. 2015b, 2017). Also, the heterogeneity of treatment protocols and the inclusion of diverse treatments in the comparison group could introduce a minor bias in the results. The findings emphasize the importance of conducting RCTs with standardized treatment protocols and long-term follow-up, as well as implementing blinding measures to enhance the validity of the results. We also recommend that future RCTs perform sample size calculations to ensure robust and reliable findings, as this will help control for alpha risk and improve the statistical power of the studies. This review serves as a solid starting point for future research to explore the efficacy of balneotherapy in the treatment of FMS, paving the way for exploring combined and personalized therapies that optimize the benefits of balneotherapy and other complementary treatments. It is crucial to focus on how to maximize the effectiveness of balneotherapy, either by modulating water properties, such as mineral concentration or by combining balneotherapy with other therapeutic approaches, such as physical exercise and psychological therapy. This integrated approach could offer FMS patients a more comprehensive and holistic symptom management.

## Conclusions

This systematic review and meta-analysis of aggregated data assessed the effectiveness of balneotherapy as therapeutic method in reducing the impact of FMS. Our findings showed that balneotherapy may be effective just to finish the intervention in reducing pain, disability and depression in patients with FMS. Besides, this meta-analysis suggests that the effectiveness of balneotherapy could be maintained 1 and 3 months since the end of the intervention on pain and disability. Finally, with caution, due to the low number of studies included (< 8), the low number of patients per meta-analysis in balneotherapy group (< 400) and the statistical heterogeneity, balneotherapy seems to be effective to maintain reduced pain, disability and depression at 6 months since the end of the intervention. The findings underscore the necessity of conducting high-quality RCTs with standardized treatment protocols, extensive long-term follow-up, and rigorous blinding measures to enhance the validity and reliability of study outcomes. This methodological rigor is essential for a comprehensive understanding of balneotherapy’s role in FMS management. Furthermore, these conclusions highlight the importance of a holistic treatment approach that addresses both the physical and psychological aspects of FMS. This systematic review advocates for further exploration into combined and personalized therapies, which could potentially amplify the overall efficacy of balneotherapy in the treatment of FMS, underscoring its role as a key component in the multifaceted management of this complex condition.

## Electronic supplementary material

Below is the link to the electronic supplementary material.

Supplementary Material 1

Supplementary Material 2

Supplementary Material 3

Supplementary Material 4

Supplementary Material 5

Supplementary Material 6

Supplementary Material 7

Supplementary Material 8

Supplementary Material 9

Supplementary Material 10

Supplementary Material 11

Supplementary Material 12
