# Supplementary material for: Effectiveness of balneotherapy in reducing pain, disability, and depression in patients with Fibromyalgia syndrome: a systematic review with meta-analysis
Source: Int J Biometeorol. 2024 Jul 15;68(10):1935–51. doi: 10.1007/s00484-024-02732-3 (PMC11493822; doi:10.1007/s00484-024-02732-3)
Supplement: Supplementary file 8 — Supplementary Material 8 [file 484_2024_2732_MOESM8_ESM.docx]

Online Resource 1. Search strategy employed in each database.

| DATABASE | SEARCH STRATEGY |
| --- | --- |
| PubMed Medline | ((("Balneology" [MeSH Terms] OR "hydrotherapy" [MeSH Terms] OR "spa therapy" [MeSH Terms] OR "water therapy" [Title/Abstract] OR "aquatic therapy" [Title/Abstract] OR "thermal therapy" [Title/Abstract] OR "mineral baths" [Title/Abstract] OR "balneotherapy" [Title/Abstract] OR "medicinal water" [Title/Abstract])) AND ("Fibromyalgia" [MeSH Terms] OR "fibromyalgia" [Title/Abstract] OR "Fibrositis" [MeSH Terms] OR "Myofascial Pain Syndromes" [MeSH Terms] OR "chronic widespread pain" [Title/Abstract])) |
| Science Direct | (("Balneology" OR "hydrotherapy" OR "spa therapy" OR "water therapy" OR "aquatic therapy" OR "thermal therapy" OR "mineral baths" OR "balneotherapy" OR "medicinal water") AND ("fibromyalgia" OR "fibrositis" OR "myofascial pain" OR "chronic widespread pain")) |
| CINAHL  Complete | (AB ("Balneology" OR "Hydrotherapy" OR "Spa Therapy" OR "Water Therapy" OR "Aquatic Therapy" OR "Thermal Therapy" OR "Mineral Baths" OR "Balneotherapy" OR "Medicinal Water") AND AB ("Fibromyalgia" OR "Fibrositis" OR "Myofascial Pain Syndromes" OR "chronic widespread pain")) |
| Scopus | (TITLE-ABS-KEY ("balneology" OR "hydrotherapy" OR "spa therapy" OR "water therapy" OR "aquatic therapy" OR "thermal therapy" OR "mineral baths" OR "balneotherapy" OR "medicinal water") AND TITLE-ABS-KEY ("fibromyalgia" OR "fibrositis" OR "myofascial pain" OR "chronic widespread pain")) |
| Web of Science | (TOPIC: ("Balneology" OR "hydrotherapy" OR "spa therapy" OR "water therapy" OR "aquatic therapy" OR "thermal therapy" OR "mineral baths" OR "balneotherapy" OR "medicinal water") AND TOPIC: ("fibromyalgia" OR "fibrositis" OR "myofascial pain" OR "chronic widespread pain")) |
