# Supplementary material for: Effectiveness of balneotherapy in reducing pain, disability, and depression in patients with Fibromyalgia syndrome: a systematic review with meta-analysis
Source: Int J Biometeorol. 2024 Jul 15;68(10):1935–51. doi: 10.1007/s00484-024-02732-3 (PMC11493822; doi:10.1007/s00484-024-02732-3)
Supplement: Supplementary file 9 — Supplementary Material 9 [file 484_2024_2732_MOESM9_ESM.docx]

**Online Resource 4.** Main Findings in Meta-Analyses.

| **Outcome** | **Time-point assessment** | **Findings Summary** | | | | | | | | | | **Quality Evidence (GRADE Assessment)** | | | | | |
| --- | --- | --- | --- | --- | --- | --- | --- | --- | --- | --- | --- | --- | --- | --- | --- | --- | --- |
|  |  | **Effect Size** | | | | | **Heterogeneity** | | **Publication Bias** | | |  |  |  |  |  |  |
|  |  | **K** | **C** | **SMD** | **95% CI** | ***p*** | **Q (df)** | **I^2^ *(p)*** | **Egger *p*** | **Trim and Fill** | | **Risk of**  **bias** | **Incons** | **Indir** | **Imprec** | **Pub Bias** | **Quality** |
|  |  |  |  |  |  |  |  |  |  | **Adj SMD (95% CI)** | **% var** |  |  |  |  |  |  |
| **Pain (VAS)** | Immediate | 12 | 12 | -1.67 | -2.18 to -1.16 | <0.001 | 20.69 (11) | 46.73% (0.04) | <0.001 | -1.8 (-2.36 to -1.25) | 8% | Med | Med | No | No | No | High |
|  | 1 month | 7 | 7 | -1.82 | -2.48 to -1.16 | <0.001 | 7.71 (6) | 21.32% (0.26) | <0.001 | -2.1 (-3 to -1.13) | 15% | Med | Low | No | Yes | Yes | Low |
|  | 3 months | 9 | 9 | -0.86 | -1.41 to -0.3 | 0.003 | 9.25 (8) | 13.53% (0.32) | 0.03 | -0.95 (-1.43 to -0.48) | 5% | Med | Low | No | Yes | No | Moderate |
|  | 6 months | 8 | 8 | -1 | -1.62 to -0.38 | 0.002 | 35.42 (7) | 67.25% (<0.01) | 0.05 | -1.4 (-2.2 to -0.59) | 40% | Med | Large | No | Yes | Yes | Low |
| **Disability (FIQ)** | Immediate | 12 | 12 | -1.1 | -1.46 to -0.7 | <0.001 | 21.52 (11) | 48.81% (0.03) | 0.01 | -1.36 (-1.8 to -0.91) | 23% | Med | Large | No | No | Yes | Low |
|  | 1 month | 6 | 6 | -0.78 | -1.31 to -0.25 | 0.004 | 5.97 (5) | 16.32% (0.31) | 0.09 | -1 (-1.52 to -0.51) | 28% | Med | Low | No | Yes | Yes | Low |
|  | 3 months | 12 | 12 | -0.8 | -1.16 to -0.43 | <0.001 | 13.76 (11) | 20.17% (0.25) | 0.04 | -0.93 (-1.29 to -0.57) | 16% | Med | Low | No | No | Yes | Low |
|  | 6 months | 6 | 6 | -0.77 | -1.29 to -0.24 | 0.004 | 8.62 (5) | 37.41% (0.13) | 0.01 | -1.2 (-2 to -0.42) | 55% | Med | Med | No | Yes | Yes | Low |
| **Depression** | Immediate | 9 | 9 | -0.51 | -0.93 to -0.9 | 0.017 | 9.76 (8) | 18.11% (0.28) | 0.74 | -0.51 (-0.93 to -0.9) | 0% | Med | Low | No | Yes | No | Low |
|  | 1 month | 4 | 4 | -0.14 | -0.76 to 0.48 | 0.654 | 3.33 (3) | 9.81% (0.35) | 0.67 | -0.14 (-0.76 to 0.48) | 0% | Med | Low | No | Yes | No | Low |
|  | 3 months | 8 | 8 | -0.07 | -0.49 to 0.36 | 0.755 | 8.74 (7) | 17.92% (0.28) | 0.74 | -0.07 (-0.49 to 0.36) | 0% | Med | Low | No | Yes | No | Low |
|  | 6 months | 5 | 5 | -0.57 | -1.12 to -0.03 | 0.040 | 4.78 (4) | 15.41% (0.31) | 0.09 | -0.25 (-0.69 to 0.17) | 100% | Med | Low | No | Yes | Yes | Low |
