# Supplementary figures and images for: Effectiveness of balneotherapy in reducing pain, disability, and depression in patients with Fibromyalgia syndrome: a systematic review with meta-analysis
Source: Int J Biometeorol. 2024 Jul 15;68(10):1935–51. doi: 10.1007/s00484-024-02732-3 (PMC11493822; doi:10.1007/s00484-024-02732-3)

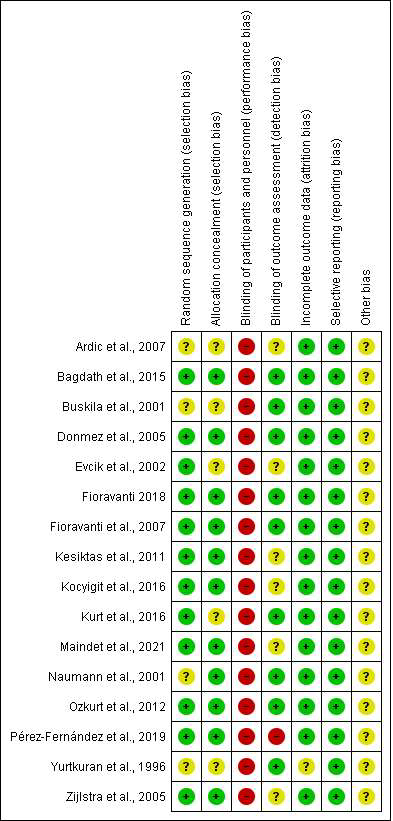

Supplement: Supplementary file 11 — Supplementary Material 11 [file 484_2024_2732_MOESM11_ESM.png]

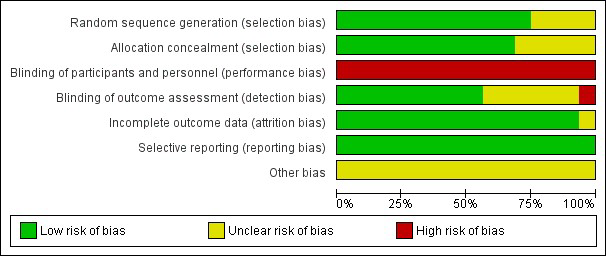

Supplement: Supplementary file 12 — Supplementary Material 12 [file 484_2024_2732_MOESM12_ESM.png]
